# *Plasmodium vivax* and human hexokinases share similar active sites but display distinct quaternary architectures

**DOI:** 10.1107/S2052252520002456

**Published:** 2020-03-26

**Authors:** Shanti Swaroop Srivastava, Joseph E. Darling, Jimmy Suryadi, James C. Morris, Mark E. Drew, Sriram Subramaniam

**Affiliations:** a University of British Columbia, Vancouver, British Columbia, Canada; b Laboratory of Cell Biology, Center for Cancer Research, National Cancer Institute, National Institutes of Health, Bethesda, MD 20892, USA; c Eukaryotic Pathogens Innovation Center, Department of Genetics and Biochemistry, Clemson University, Clemson, South Carolina, USA; d Department of Microbial Infection and Immunity, The Ohio State University, Wexner Medical Center, Columbus, Ohio, USA

**Keywords:** malaria, *Plasmodium vivax*, cryo-EM, hexokinase

## Abstract

Malaria is a devastating disease caused by a protozoan parasite. It affects over 300 million individuals and results in over 400 000 deaths annually, most of whom are young children under the age of five. Hexokinase, the first enzyme in glucose metabolism, plays an important role in the infection process and represents a promising target for therapeutic intervention. Here, cryo-EM structures of two conformational states of *Plasmodium vivax* hexokinase (PvHK) are reported at resolutions of ∼3 Å. It is shown that unlike other known hexokinase structures, PvHK displays a unique tetrameric organization (∼220 kDa) that can exist in either open or closed quaternary conformational states. Despite the resemblance of the active site of PvHK to its mammalian counterparts, this tetrameric organization is distinct from that of human hexokinases, providing a foundation for the structure-guided design of parasite-selective antimalarial drugs.

## Abstract 2

A few successful drugs are available against malaria, but the magnitude of the impact caused by this disease requires additional measures for successful treatment and the goal of eradication. Here, new information is presented on hexokinase, a key protein in the infection process that represents a promising target for therapeutic intervention.

## 1. Introduction   

Malaria is a devastating disease that ravages large parts of the developing world. It infects millions of individuals every year and kills hundreds of thousands, most of whom are children under the age of five (World Health Organization, 2018 ▸). Malaria is caused by protozoan parasites of the genus *Plasmodium*, of which *P. falciparum* and *P. vivax* are the two predominant species associated with disease mortality and morbidity (Naing *et al.*, 2014 ▸; World Health Organization, 2018 ▸). Additionally, *P. vivax*, the most widespread species, can reside in undetectable, dormant liver stages called hypnozoites that can become activated months after infection and result in recurrent disease. Only a single class of drug (8-aminoquinolines), the mode of action of which is not well understood, is active against hypnozoites, and its use is complicated and potentially lethal to the patient (Wells *et al.*, 2010 ▸). During the intra-erythrocytic stage of the parasite, which causes pathogenesis in humans, glucose metabolism via glycolysis plays a critical metabolic role for the parasite (Alam *et al.*, 2014 ▸; Preuss *et al.*, 2012 ▸; Vaidya & Mather, 2009 ▸). Glucose consumption is up to 100-fold higher in erythrocytes infected with parasites (Roth *et al.*, 1982 ▸) and knockout of the parasite glucose transporter appears to be lethal (Slavic *et al.*, 2010 ▸). In addition, glycolysis inhibitors deplete parasite ATP (van Schalkwyk *et al.*, 2008 ▸). Taken together, these results strongly indicate that glycolysis is vital for parasite growth and thus components of the glycolytic pathway should be considered as promising targets for novel therapeutics (Alam *et al.*, 2014 ▸; Preuss *et al.*, 2012 ▸). Consistent with this hypothesis, many glucose analogs have been shown to have antiparasitic activity (Santos de Macedo *et al.*, 2001 ▸; Udeinya & Van Dyke, 1981 ▸; van Schalkwyk *et al.*, 2008 ▸).

Hexokinases (HKs) are enzymes that are responsible for carrying out the first step in glycolysis: the phosphorylation of glucose to glucose 6-phosphate (G6P). In *Plasmodium*, the G6P that results from HK activity is either further metabolized in the glycolytic pathway to produce ATP or funneled into the pentose phosphate pathway to generate NADPH and ribulose 5-phosphate, which are used in antioxidant defense and for *de novo* nucleotide triphosphate synthesis, respectively (Atamna *et al.*, 1994 ▸; Becker *et al.*, 2004 ▸; Müller, 2004 ▸). Hexokinase activity in infected erythrocytes is 25-fold higher compared with uninfected erythrocytes, which is in agreement with the increased consumption of glucose by infected erythrocytes (Roth, 1987 ▸). Although the *Plasmodium* hexokinases are well conserved within the genus (∼90% identity), they share little sequence identity with mammalian HKs outside the essential ATP- and hexose-binding pockets. For example, *P. vivax* hexokinase (PvHK) and *P. falciparum* hexokinase (PfHK) share only 26–32% sequence identity with human hexokinases (Olafsson *et al.*, 1992 ▸; Harris *et al.*, 2013 ▸; Supplementary Fig. S1). Monoclonal or polyclonal antibodies against PfHK do not cross-react with mammalian hexokinases, indicating that these parasite-derived hexokinases are antigenically distinct from their mammalian homologs (Olafsson *et al.*, 1992 ▸). Interestingly, it has recently been reported that PvHK can be detected by RNA sequencing using an *in vitro* hypnozoite model (Gural *et al.*, 2018 ▸). If this finding is representative of *in vivo* hypnozoites, it would suggest a role for PvHK in these hard-to-treat stages of the disease. Additionally, inhibitors of PfHK have been identified in high-throughput screens that show no impact on the human counterpart enzyme, suggesting that structural differences exist that can be exploited for selective drug design (Davis *et al.*, 2016 ▸). Here, we describe cryo-EM structures of PvHK at ∼3 Å resolution, documenting its novel tetrameric organization and conformational variability. This provides new insights into similarities and differences between the parasite and mammalian hexokinases that could be critical for the rational design of parasite-selective small-molecule HK inhibitors.

## 2. Methods   

### 2.1. Protein production   

*P. vivax* hexokinase (PvHK; UniProt ID A5K274) was expressed in *Escherichia coli* Origami 2 cells using a codon-optimized open reading frame cloned into a pQE-30 expression vector (Qiagen, Valencia, California, USA). Briefly, protein expression in the transformed *E. coli* cells was induced using 0.5 m*M* isopropyl β-d-1-thiogalactopyranoside (IPTG) when the OD_600_ of the culture reached ∼0.6 and the cells were subsequently grown overnight at 25°C. The expressed protein was purified using the protocol described previously for PfHK (Davis *et al.*, 2016 ▸). Briefly, the cell pellets were resuspended in lysis buffer (50 m*M* Tris–HCl pH 8, 300 m*M* NaCl, 20 m*M* imidazole) supplemented with 10 m*M* glucose, 0.1% Triton X-100, 1 mg ml^−1^ lysozyme, 2.5 m*M* MgCl_2_, 12.5 µg DNAse I, 0.5 m*M* CaCl_2_ and one protease-inhibitor tablet (EDTA-free, Thermo Fisher Scientific, Waltham, Massachusetts, USA) per litre of culture and lysed by sonication. The cleared lysate was then applied onto a HisTrap Crude FF column pre-equilibrated with 25 m*M* imidazole in column buffer (20 m*M* Tris–HCl pH 8, 150 m*M* NaCl). Following washing, the protein was eluted with column buffer containing 250 m*M* imidazole. The pooled active fractions were dialyzed into modified wash buffer (20 m*M* Tris–HCl pH 8, 50 m*M* NaCl, 1 m*M* DTT) and the sample was loaded onto a HiTrap Q XL column pre-equilibrated with modified wash buffer (buffer *A*; 20 m*M* Tris–HCl pH 8). Following extensive washes with modified wash buffer, the protein was eluted with a gradient of NaCl using buffer *B* (20 m*M* Tris–HCl pH 8, 1 *M* NaCl), with PvHK eluting at ∼45% buffer *B*, and the purity of the active fractions (as identified by enzyme assay) was assessed to be >99% on a Coomassie-stained SDS–PAGE gel. The protein was dialyzed against 2× cryo buffer (40 m*M* Tris–HCl pH 7.5, 100 m*M* NaCl, 2 m*M* TCEP) using 10 kDa molecular-weight cutoff dialysis tubing. The protein was concentrated using a Sartorius Vivaspin Turbo 15 (Göttingen, Germany) centrifuge at 4000*g* until the protein concentration reached 10 mg ml^−1^.

### 2.2. Cryo-EM specimen preparation   

PvHK at a concentration of 5 mg ml^−1^ in buffer [20 m*M* Tris pH 7.5, 50 m*M* NaCl, 1 m*M* tris(2-carboxyethyl)phosphine (TCEP)] was applied to Quantifoil Cu 200 mesh grids (1.2/1.3) that were plasma cleaned using a Solarus plasma cleaner (Gatan). The sample was blotted for 3 s and plunge-frozen in liquid ethane cooled by liquid nitrogen using an FEI Vitrobot plunge-freezing instrument. The blotting chamber was maintained at 20°C and a humidity of 100%. Cryo-EM imaging was carried out with an FEI Titan Krios operating at 300 kV. Images were acquired with a K2 Summit camera placed at the end of a Gatan Imaging Filter (GIF) in super-resolution mode with a magnified pixel size of 0.4177 Å. Images were collected spanning a defocus range of 0.5–3 µm. The dose rate was ∼1.8 e^−^ per pixel per second and the exposure time was 23.2 s. Movies were collected at a rate of 2.5 frames per second, giving 58 frames per image.

### 2.3. Data processing   

3D reconstruction was carried out using *RELION*-3.0 (Zivanov *et al.*, 2018 ▸). Alignment of movie frames and motion correction were carried out using *MotionCor*2 (Zheng *et al.*, 2017 ▸) and CTF determination was performed with *CTFfind* (Rohou & Grigorieff, 2015 ▸). Images from vitrified specimens displayed a broad distribution of orientations [Supplementary Fig. S2(*a*)]. Initially, around ∼800 particles were selected manually and the 2D class averages generated were used iteratively for automated particle selection. The particles were re-extracted with a box size of 512 pixels and a binning factor of 2. Multiple rounds of 2D classification were performed to select the particles that contributed to the highest resolution, and the resulting 2D class averages displayed distinct views with visible secondary-structural elements [Supplementary Fig. S2(*b*)]. A total of 24 390 particles with an average pixel size of 0.8354 Å were used for 3D model building and refinement. The initial model was subjected to 3D classification into four classes using local grid search and *D*2 symmetry. The two major classes (class 2 with 12 604 particles and class 4 with 6623 particles) were selected for further refinement. Class 2 and class 4 were subjected to CTF refinement and Bayesian polishing before postprocessing (Zivanov *et al.*, 2019 ▸). The overall resolution was estimated in the postprocessing step using a gold-standard Fourier shell correlation (FSC) at 0.143. Finally, two 3D classes were used for interpretation of PvHK: the major class (class 2, state I) with 3.3 Å resolution and a local higher resolution of ∼3.0 Å [Supplementary Figs. S2(*c*) and S2(*d*)], and a second class (class 4, state II) with 3.5 Å resolution and a local higher resolution of ∼3.3 Å [Supplementary Figs. S2(*e*) and S2(*f*)].

### 2.4. Model fitting and refinement   

Starting models for PvHK were prepared using *SWISS-MODEL* (Waterhouse *et al.*, 2018 ▸) based on crystal structures of *Arabidopsis* hexo­kinase 1 (AtHXK1), which has 33% sequence identity and a sequence coverage of 87% compared with PvHK, in the ligand-free form (PDB entry 4qs8) and a glucose-bound form (PDB entry 4qs7) (Feng *et al.*, 2015 ▸). The models were fitted as rigid bodies into the respective maps using *UCSF Chimera* (Pettersen *et al.*, 2004 ▸). The tetramer was generated using the *phenix.apply_ncs* module in *Phenix* and was used as input for real-space refinement in *Phenix* with the morphing and simulated-annealing options activated (Liebschner *et al.*, 2019 ▸). The output coordinate files were manually improved in *Coot* using the cryo-EM density map as a guide (Emsley *et al.*, 2010 ▸). In both states, the small domain has poor density compared with the large domain. In state I, the N-terminal tag and regions corresponding to residues 1–16, 131–151, 171–175, 200–212 and 488–493 were not modeled owing to weak density. Similarly, in state II the N-terminal tag and residues from segments 1–17, 131–144, 167–177, 227–235 and 486–493 could not be modeled. Side chains were manually refined in a single protomer and the coordinates for the tetramer were generated for refinement using commands in *Phenix*. The figures were rendered using *UCSF Chimera* (Pettersen *et al.*, 2004 ▸), *ChimeraX* (Goddard *et al.*, 2018 ▸) and *PyMOL* (version 2.0, Schrödinger). Sample-preparation, data-collection and refinement statistics are given in Table 1 ▸.

## 3. Results and discussion   

### 3.1. Overall structure of PvHK   

**Figure 1:**
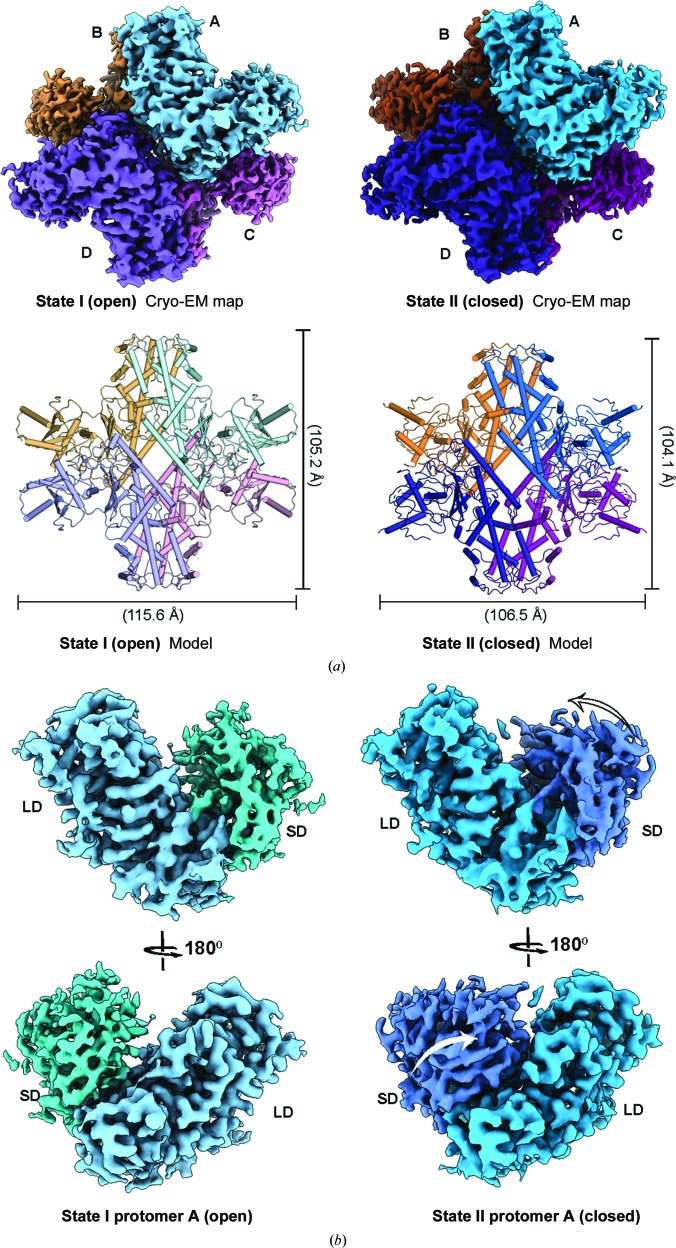
Overall structure of PvHK. (*a*) Cryo-EM structures of PvHK in the open (3.3 Å resolution) and closed (3.5 Å resolution) states. The corresponding models are shown in ribbon representation. The structure reveals that PvHK exists as a tetramer, with four protomers arranged with *D*2 symmetry. (*b*) Cryo-EM structures of a protomer from both the open and closed states are shown. The small domain (SD) and large domain (LD) of the protomers are highlighted with distinct colors.

To aid in the effort to develop parasite-selective HK inhibitors, we sought to understand the structural differences between human and *Plasmodium* hexokinases. The cryo-EM structure of PvHK demonstrates that PvHK is a tetramer with *D*2 symmetry [Fig. 1 ▸(*a*), Supplementary Fig. S3(*a*)]. Two distinct quaternary conformations are observed: state I and state II. The PvHK protomer has a domain architecture similar to those of other hexokinases, with a two-domain arrangement comprising a large domain and a small domain [Fig. 1 ▸(*b*), Supplementary Fig. S3(*a*)] (Kuettner *et al.*, 2010 ▸; Feng *et al.*, 2015 ▸; Whittington *et al.*, 2015 ▸). In both conformations the density for the small domain is weak, suggesting that this region is more flexible than the large domain [Fig. 1 ▸, Supplementary Figs. S2(*c*) and S2(*e*)]. Comparison of the structures derived for these conformational states shows that state I matches the ‘open’ state observed for hexokinases [Fig. 1 ▸, Supplementary Fig. S3(*a*)]. In contrast, state II matches the ‘closed’ state observed for hexokinases bound to either glucose or other ligands in addition to glucose [Fig. 1 ▸, Supplementary Fig. S3(*a*)] (Bennett & Steitz, 1980 ▸). Notably, a glucose-bound open state has been observed previously in *Kluyveromyces lactis* hexokinase (PDB entry 3o5b; Kuettner *et al.*, 2010 ▸). We conclude that conformational states I and II therefore correspond to the open and closed conformations of PvHK, respectively (Fig. 1 ▸). Superposition of the two states of tetrameric PvHK (*SSM* superposition in *Coot*) results in an r.m.s.d. of 2.4 Å over 1484 C^α^ atoms, suggesting significant changes.

### 3.2. Comparison of the active sites of human hexokinase IV and PvHK   

**Figure 2:**
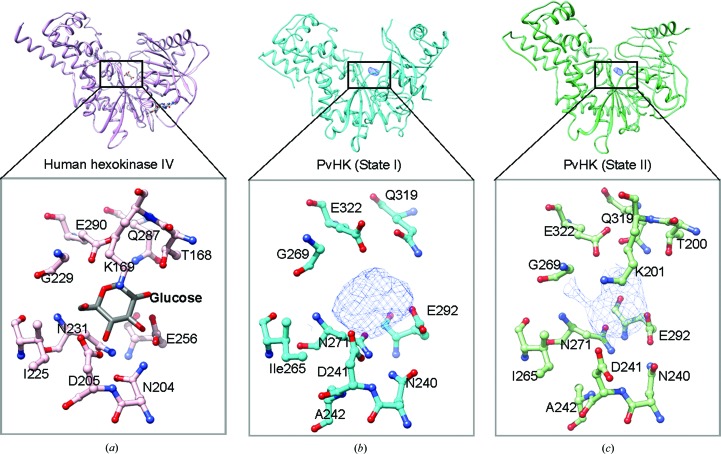
Comparison of the active sites of human hexokinase IV and PvHK. (*a*) Crystal structure of human hexokinase IV (glucokinase) in the active closed conformation with BGC (glucose) and activator. The glucose-binding site is highlighted in the close-up view and the glucose-coordinating residues are shown (PDB entry 4dhy). (*b*) Cryo-EM-derived PvHK protomer in the open state. The unmodeled ligand density (mesh) at the hexose-binding site is shown along with the residues that appear to coordinate the ligand. (*c*) The PvHK protomer in the closed conformation with extra density present in the active site. In the close-up view, the extra density and coordinating residues are shown for a one-to-one comparison with human hexokinase. Most of the residues in human hexokinase and PvHK are conserved and are seen in a very similar conformation in PvHK to that observed in human HKs.

In the cryo-EM structures of PvHK there is extra density in both the open-state and closed-state conformations that corresponds closely to the site where glucose is expected to be located based on comparison with X-ray structures of glucose-bound hexo­kinases (Fig. 2 ▸). We cannot be certain that the bound ligand in the PvHK structure is indeed glucose, as other similar sugars are present during protein expression inside the cells. However, glucose is the most likely ligand given that 10 m*M* glucose was present during cell lysis and there is an excellent correspondence between the residues that appear to interact with the observed extra density and the corresponding residues that interact with glucose in the X-ray structures of glucose-bound hexokinases (Fig. 2 ▸). For example, in human hexokinase IV the active-site residues Thr168, Lys169, Asn204, Asp205, Glu256, Glu290 and Gly229 interact with a glucose molecule (Liu *et al.*, 2012 ▸). Similarly, in PvHK the corresponding residues are Thr200, Lys201, Asn240, Asp241, Glu292, Glu322 and Gly269 [Fig. 2 ▸(*a*)]. Thus, all of the conserved residues in PvHK are proximal to the observed ligand density. These comparisons indicate that the substrate-binding sites of the gluco­kinases from *P. vivax* and human are highly conserved.

### 3.3. Molecular interactions of the PvHK tetramer   

**Figure 3:**
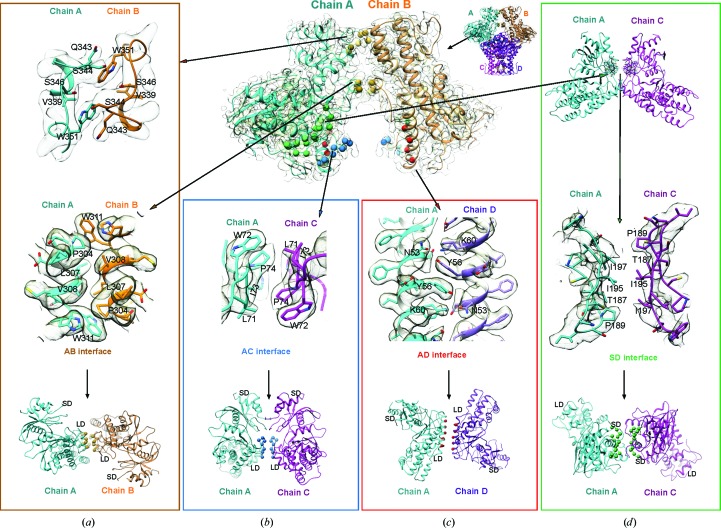
Molecular interactions of the PvHK tetramer. Four distinct interfaces are highlighted by coloring C^α^ atoms as golden spheres for residues in the AB interface, blue spheres for the AC interface, red spheres for the AD interface and green spheres for the SD interface. The interatomic interactions at the three interfaces between large domains are shown for the (*a*) AB, (*b*) AC and (*c*) AD interfaces. (*d*) The SD interface between PvHK small domains. Insets show the interactions at the residue level along with the cryo-EM map.

In the PvHK tetramer the large domain makes the major inter-protomer contacts, whereas the small domain is mostly solvent-exposed. Most of the interfacial interactions are stacking and hydrophobic in nature. Three distinct regions from the large domain form the major interfaces for the inter-protomer interactions that are involved in the tetrameric assembly. The first major interface is formed by the region corresponding to the helix spanning Pro304–Trp311 and the segment Val339–Trp351. Pro304, Leu307, Val308 and Trp311 of this segment from protomers A and B interact with each other across a twofold axis, forming a strong hydrophobic interaction [Fig. 3 ▸(*a*)]. The interaction between protomers A and B is further enhanced by the hydrophobic interaction of Val339 and Trp351 from both protomers and hydrogen bonds formed from Ser344 and Lys352 to Trp351 and Glu28, respectively, from neighboring protomers [Fig. 3 ▸(*b*)]. We refer to this interface as the ‘AB interface’. Similar interactions are formed between protomers C and D since the tetramer has *D*2 symmetry. The second interface is formed by the segment between Asn70 and His75 along a different twofold axis. We refer to this as the ‘AC interface’, in which Leu71, Trp72, Ile73 and Pro74 from opposing protomers form hydrophobic interactions [Fig. 3 ▸(*b*)]. Importantly, compared with human hexokinases, this segment is an insertion in PvHK and forms critical interactions for tetramer formation. The third interface is the helix between Tyr44 and Arg66, which interacts along a third twofold axis. We refer to this as the ‘AD interface’, in which the Tyr56 residues from chains A and B are positioned over the Tyr56 residues from chains D and C, respectively [Fig. 3 ▸(*c*)]. In addition, many polar residues in this interface interact with each other. For example, residues Lys60 and Asn53 from chains A and B form hydrogen bonds to residues Asn53 and Lys60 from chains D and C, respectively. Compared with the large domain, the small domain forms minimal contacts and we refer to the interface between two small domains as the ‘SD interface’. Owing to the flexible nature of the small domain, the inter-protomer contacts between the small domains are not resolved at high resolution. However, it is apparent from the structure that the contacts at the SD interface are formed by hydrophobic interactions of residues Thr187, Pro189, Ile195 and Ile197 from both protomers [Fig. 3 ▸(*d*)]. Other residues which are polar in nature seem to further enhance the contacts at the SD interface, but owing to the poor resolution of the side chains we cannot be certain about the specific nature of these interactions.

### 3.4. Structural comparison between PvHK conformational states   

**Figure 4:**
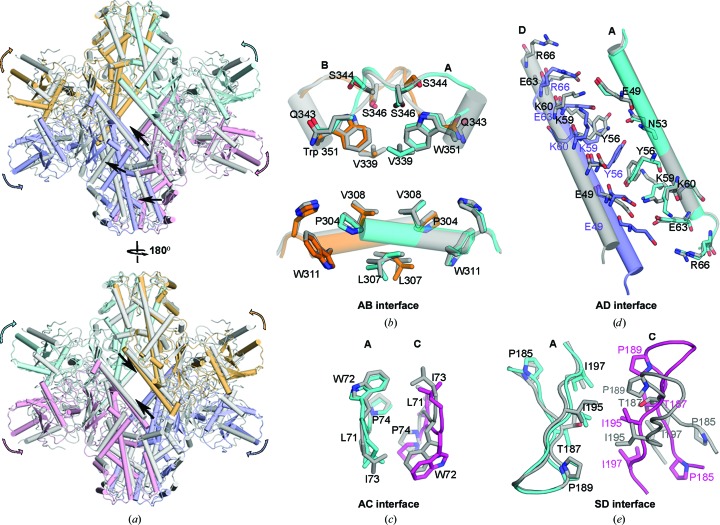
Structural comparison between PvHK conformational states. (*a*) Superposition of open (I) and closed (II) states of the PvHK tetramer. Open-state protomers are shown in four different colors, while the corresponding protomers in the closed state are shown in gray. Superposition of the residues at the (*b*) AB, (*c*) AC, (*d*) AD and (*e*) SD interfaces highlight the relative changes in the open (colored) and closed (gray) states.

The description above is based on the open conformational state, but the same interfaces also form the major inter-protomer contacts in the closed state, but with some important differences [Fig. 4 ▸(*a*)]. While there is almost no change in the interactions at the major AB interface, which is dominated by strong hydrophobic contacts within the tetramer [Fig. 4 ▸(*b*), Supplementary Fig. S3(*b*)], the AC interface interactions made via segment 2 appear to be stronger, as the distance between participating residues is slightly reduced [Fig. 4 ▸(*c*)]. Major changes are observed in the AD interface interactions, which are dominated by polar residues [Fig. 4 ▸(*d*)]. For example, residue Tyr56 that forms a stacking interaction at the AD interface moves away from the interface in state 2 compared with state 1. Also, residues Lys59 and Glu49 form hydrogen bonds to Glu49 and Lys59, respectively, from the opposite protomer. As a result of these changes, the two helices are shifted by ∼5 Å between the two states. It appears likely that the conformational changes that occur during the transition from the open to closed conformation are important for PvHK function. The transition between the two states is likely to involve rotation of the small domain (∼12°) relative to the large domain that leads to a relative rotation of ∼24° between the two small domains and a change in inter-protomer contacts at the SD interface [Fig. 4 ▸(*e*), Supplementary Fig. S3(*c*)]. In both states, density for the insertions compared with human HKs, such as LLDKHA, GRATN and KGKT, that are part of the small domain are not visible, presumably owing to disorder (Supplementary Fig. S1). Nevertheless, our results show that the PNLWIPH insertion in the large domain, which is well resolved in cryo-EM density maps, participates in the AC interface interactions that stabilize the tetramer [Fig. 3 ▸(*b*), Supplementary Fig. S1].

### 3.5. Comparison of PvHK with human hexokinase   

Evaluation of the oligomeric states of all of the hexokinases reported in the PDB shows that most hexokinases are either monomeric or dimeric (Aleshin *et al.*, 1998 ▸; Kuettner *et al.*, 2010 ▸; Nishimasu *et al.*, 2007 ▸; Rosano *et al.*, 1999 ▸; Supplementary Fig. S4). Human hexokinases I, II and III exist in monomeric or dimeric forms, but the full-length protein has two hexokinase modules as a result of gene duplication. Thus, in the dimeric arrangement of hexokinase I or II there are four kinase modules in the dimeric assembly (Aleshin *et al.*, 1998 ▸; Rosano *et al.*, 1999 ▸; Supplementary Fig. S4). However, the relative orientations of these four modules are very different when compared with PvHK. Human hexokinase IV, which is also known as glucokinase, is comparable in protomer size to PvHK, but exists as a monomer (Liu *et al.*, 2012 ▸; Whittington *et al.*, 2015 ▸) [Supplementary Fig. S4(*a*)]. The only hexokinase for which a structure has been determined so far that forms a homotetramer is that from the archaeon *Thermus thermophilus*, which is smaller in size (∼35 kDa) and is classified as an ROK (repressor, open reading frame, kinase) hexo­kinase (Nakamura *et al.*, 2012 ▸) [Supplementary Fig. S4(*f*)]. The tetrameric interactions in the archaeal hexokinase are completely different to those observed within PvHK. Thus, the tetrameric arrangement observed in PvHK is unique and distinct from those observed in all other reported hexokinase structures.

**Figure 5:**
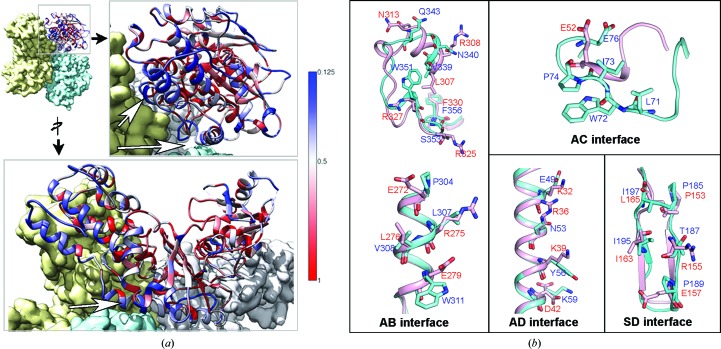
Comparison of PvHK with human hexokinase. (*a*) Sequence conservation of PvHK (compared with all human hexokinases). The residues facing the tetrameric interface are less conserved (blue). (*b*) Structural comparison between PvHK (cyan) and human hexokinase IV (pink) at each inter-protomer interface, highlighting distinct residues that mediate tetrameric contacts in PvHK.

The N- and C-terminal halves of human hexokinases I–III and human hexokinase IV share ∼26–32% sequence identity with PvHK, but their active sites are highly conserved (Supplementary Fig. S1). When the percentage sequence identity among them is mapped onto the PvHK structure, the largest differences are observed in the peripheral regions of the PvHK protomer, particularly in the inter-protomer interface region [Fig. 5 ▸(*a*)]. Several of the critical residues involved in strong hydrophobic interactions in the inter-protomer interfaces of PvHK are replaced by polar residues in human hexokinase, suggesting a structural basis for the differences in quaternary architecture [Fig. 5 ▸(*b*)]. Also, Tyr56, which is involved in an important stacking interaction in PvHK, is replaced by lysine in human hexokinase IV (Lys, Gly, Ala or Val in human hexokinases I–III) [Fig. 5 ▸(*b*), Supplementary Fig. S1]. Interestingly, these regions of PvHK are also quite distinct in other hexokinases.

## 4. Conclusion   

Malaria afflicts hundreds of millions of people every year. A few successful drugs are available (World Health Organization, 2018 ▸), but the magnitude of the impact caused by this disease requires additional measures for successful treatment and the goal of eradication. Many *Plasmodium* proteins associated with diverse key metabolic processes are already being explored as drug targets (Kumar *et al.*, 2018 ▸). Given its essential role in glucose metabolism, *Plasmodium* hexokinase has also been considered as a promising drug target (Davis *et al.*, 2016 ▸; Harris *et al.*, 2013 ▸). Small-molecule libraries have been screened against *Plasmodium* hexokinase, and inhibitory molecules have been identified that inhibit the enzyme and parasite growth, and have limited toxicity toward mammalian cells (Davis *et al.*, 2016 ▸; Harris *et al.*, 2013 ▸). Our study defining the structural differences between the parasite and human hexokinases suggest that *Plasmodium* hexokinase can be exploited as an antimalarial drug target. The unique quaternary organization observed in PvHK may serve as a means of regulating enzyme activity, which we are currently investigating. This differs from the situation in humans, in which the two-module architecture of human hexokinases I–III is thought to have arisen from gene duplication, providing regulatory mechanisms (Cárdenas *et al.*, 1998 ▸). Our structural insights have the potential to aid in the identification of novel classes of molecules that could function as allosteric inhibitors of PvHK activity.

## 5. Related literature   

The following references are cited in the supporting information for this article: Corpet (1988 ▸) and Robert & Gouet (2014 ▸).

## Supplementary Material

EMDB reference: PvHK State I (open), EMD-21458

EMDB reference: PvHK State II (closed), EMD-21459

PDB reference: PvHK State I (open), 6vyf

PDB reference: PvHK State II (closed), 6vyg

Supplementary Figures. DOI: 10.1107/S2052252520002456/fq5011sup1.pdf

## Figures and Tables

**Table 1:** Sample-preparation, data-collection and refinement statistics

Protein concentration (mg ml^−1^)	5	
Sample volume for EM grid (µl)	2.5	
Grid type	Quantifoil Cu 200 1.2/1.3	
Plunge freezer	Vitrobot Mark IV	
Blotting time (s)	3	
Blotting temperature (°C)	4	
Blotting-chamber humidity (%)	100	
Microscope	Titan Krios, Thermo Fisher (FEI)	
Camera	K2 Summit (Gatan)	
Energy filter	Yes	
*C*_s_ corrector	No	
Magnification	165000	
Dose rate (e^−^ per pixel per second)	1.8	
Dose rate (e^−^ Å^−2^ s^−1^)	2.6	
Total dose (e^−^ Å^−2^)	60.32	
No. of frames	58	
Physical pixel size (microscope) (Å per pixel)	0.8354	
Super-resolution pixel size (microscope) (Å per pixel)	0.4177	
Final pixel size used for final maps (Å per pixel)	0.8354	
Total No. of micrographs	1199	
No. of selected micrographs	1091	
No. of particles picked	181611	
No. of particles selected	24390	
Symmetry imposed	*D*2	
	State I	State II
No. of particles	12604	6623
Average resolution (Å)	3.3	3.5
FSC threshold	0.143	0.143
Model composition	Polypeptide	Polypeptide
Protein residues	1728	1732
CC_mask_	0.87	0.84
R.m.s. deviations		
Bond lengths (Å)	0.005	0.004
Bond angles (°)	0.668	0.681
Validation		
*MolProbity* score	1.95	1.96
Clashscore (all atoms)	8.15	7.99
Poor rotamers (%)	0.0	0.54
Ramachandran		
Favored (%)	91.27	90.76
Allowed (%)	8.73	9.24
Disallowed (%)	0.0	0.0
