# Supplementary material for: Plasmodium vivax and human hexokinases share similar active sites but display distinct quaternary architectures
Source: IUCrJ. 2020 Mar 26;7(Pt 3):453–61. doi: 10.1107/S2052252520002456 (PMC7201273; doi:10.1107/S2052252520002456)
Supplement: Supplementary file 1 [file m-07-00453-sup1.pdf]

IUCrJ

Volume 7 (2020)

Supporting information for article:

Plasmodium vivax and human hexokinases share similar active sites but display distinct quaternary architectures

Shanti Swaroop Srivastava, Joseph E. Darling, Jimmy Suryadi, James C. Morris, Mark E. Drew and Sriram Subramaniam

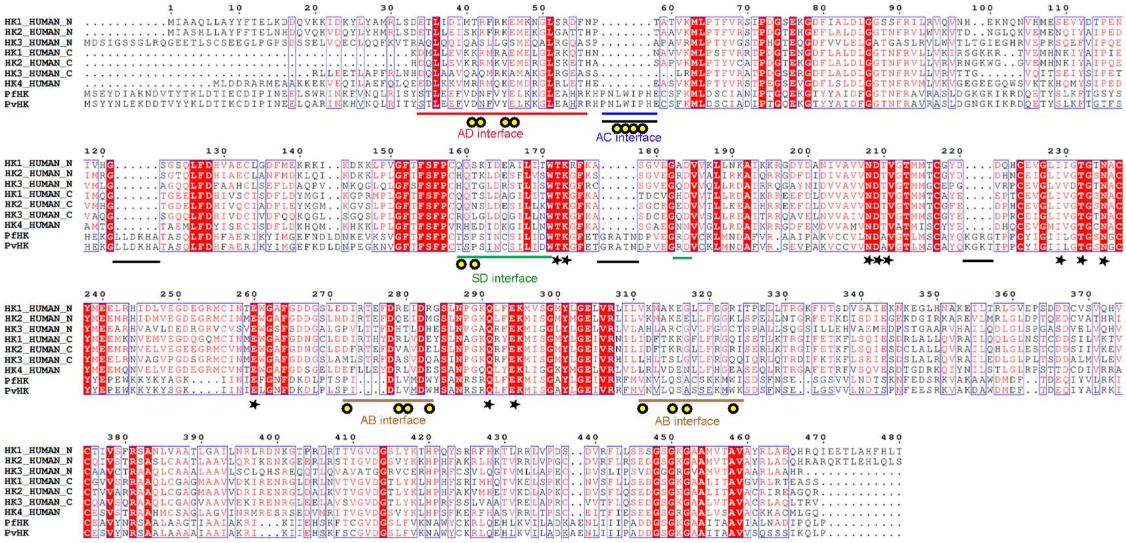

Figure S1 (A) Sequence alignment of PvHK against human hexokinases HK I, HK II HK III and HK IV (Glucokinase). N and C refers to N-terminal half and C-terminal half in huam HK I-III, respectively. Sequence alignment was done using MultAlin and rendered using EsPript (Corpet, 1988; Robert & Gouet, 2014). Residues marked with a star participate in the hexose-binding site. A black underline highlights the insertion regions. Colored underlines highlight the stretches that participate in tetrameric interactions. Yellow circles highlight residues at the interface in the PvHK tetramer.

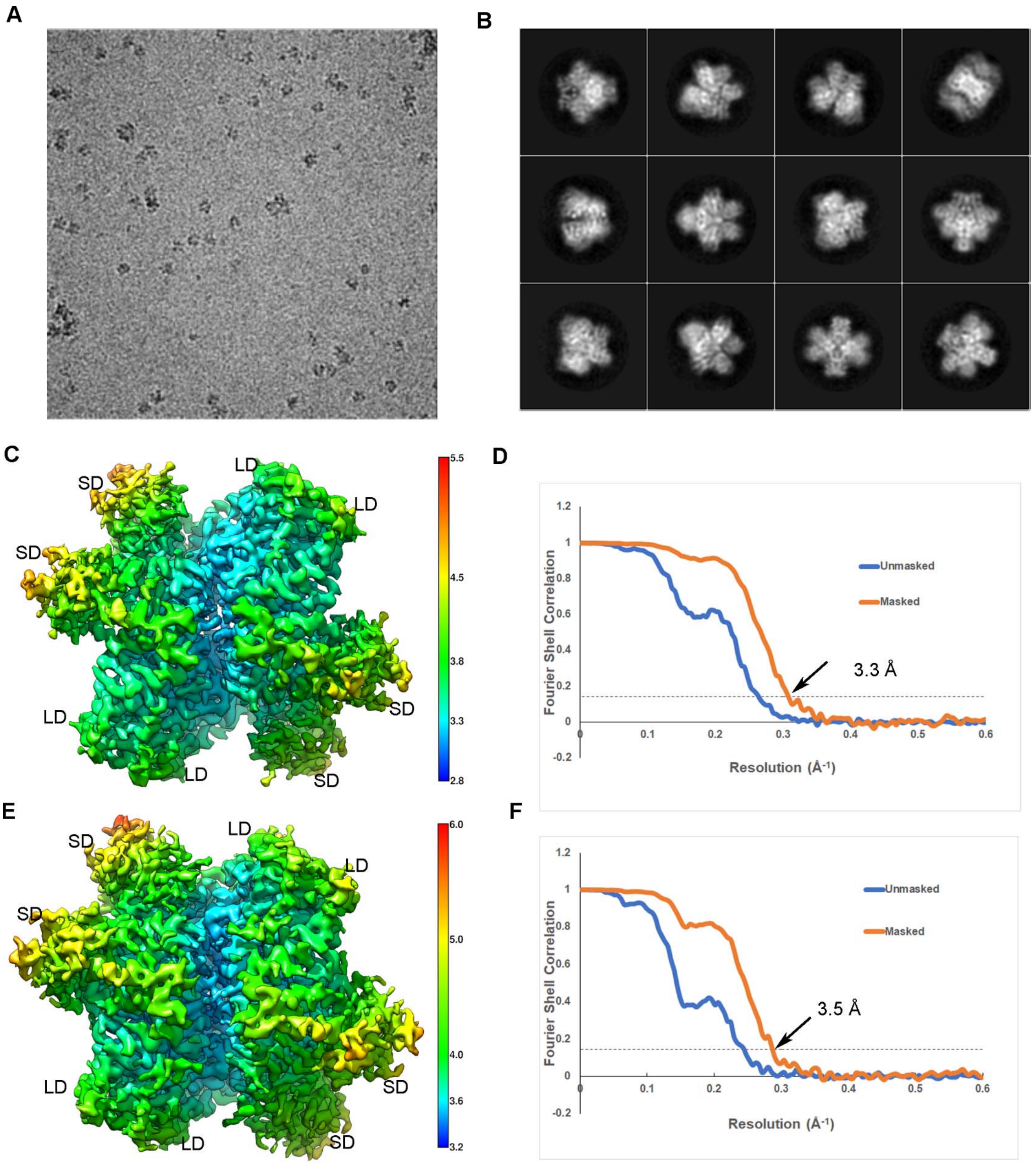

Figure S2 Overview of key steps in the cryo-EM analysis of PvHK. (A) Representative micrograph of PvHK in vitreous ice. The image was recorded at a defocus of 2.6 microns (B) Selected 2D class averages of PvHK showing the different orientations of the PvHK tetramer. (C) Local resolution map of PvHK state I obtained after postprocessing in RELION. (D) FSC plot of the PvHK state I map indicates a value of 0.143 at ~3.3 Å resolution. (E) Local resolution map of PvHK state II and (F) its FSC plot indicates a value of 0.143 at ~3.5Å.

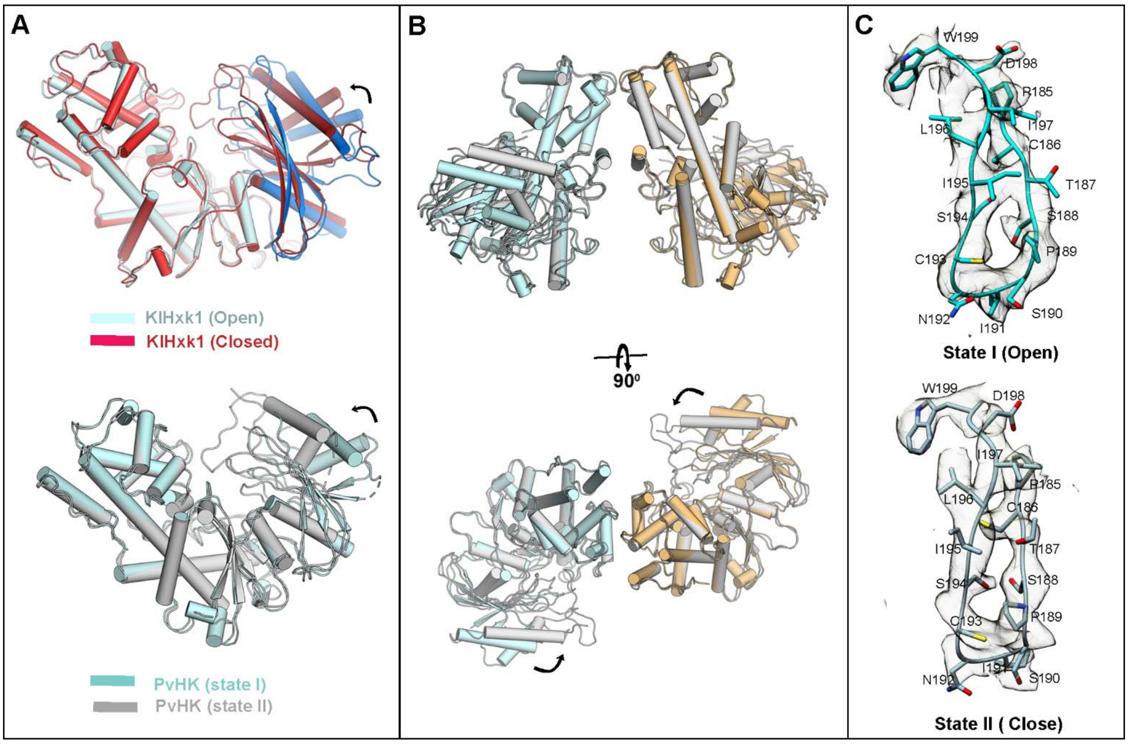

Figure S3 (A) Structural superposition of *Kluyveromyces lactis* hexokinase KlHxk1 in the open conformation (Cyan, PDB ID: 4JAX) and glucose-bound closed conformation (Red, PDB ID: 3O8M). The small domain is highlighted in blue (open state) and maroon (closed state). Superposition of a protomer of PvHK in the state I (in cyan) and state II (gray) is shown below for comparison. (B) Superposition of two PvHK protomers associating via the AB interface in state I (coloured) and state II (grey). There is almost no change in AB interface interactions in the large domain of both states. (C) Cryo-EM map of residues 185-199 from small domain of PvHK in open and closed states.

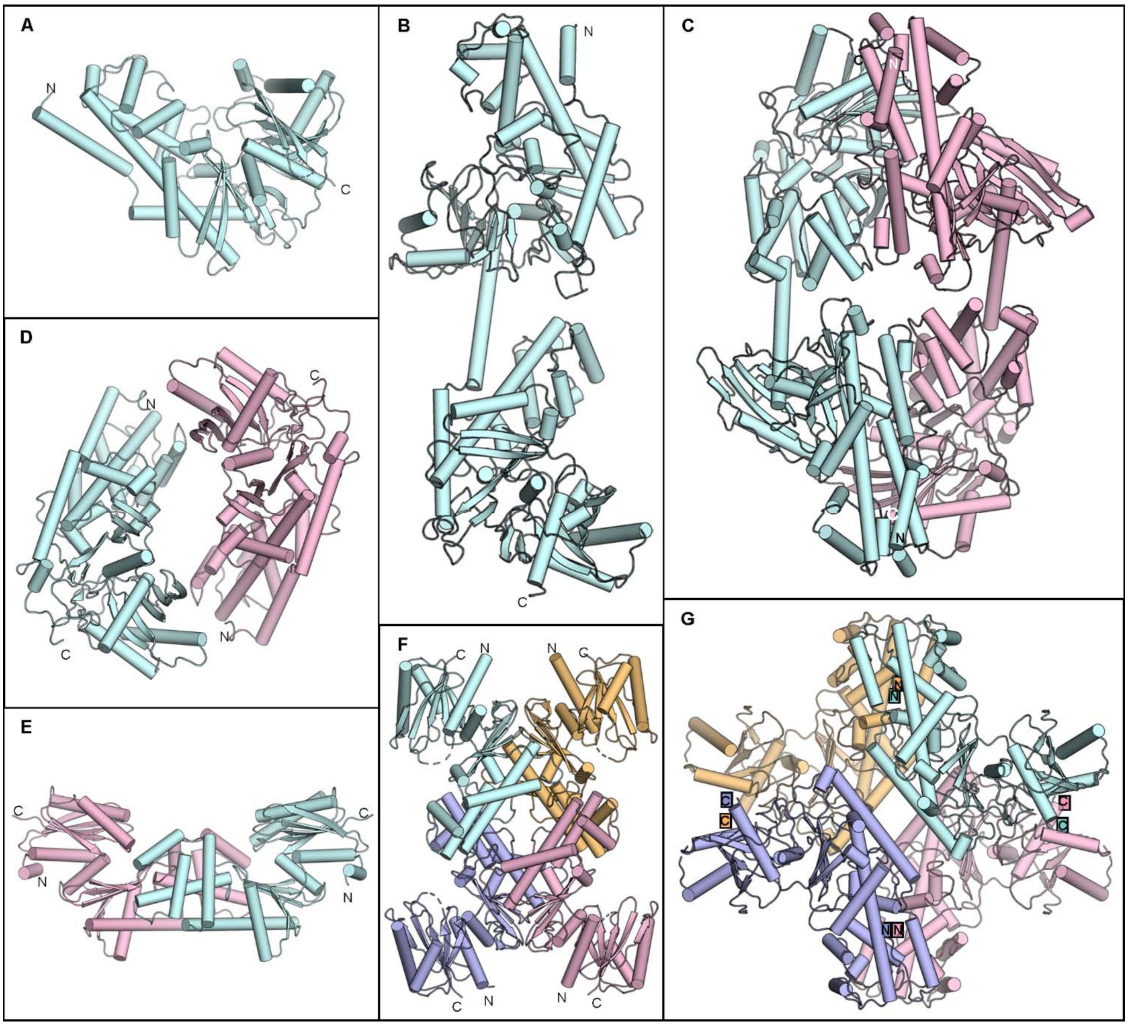

Figure S4 (A) Schematic representation of the known quaternary organization of different hexokinases. (A) Monomeric human hexokinase IV/glucokinase (PDB ID: 1V4S) (B) Monomeric human hexokinase I (PDB ID: 1HKB), (C) Concentration-dependent dimer formed by human hexokinase I (PDB ID: 4FPB), (D) Dimeric *Kluyveromyces lactis* hexokinase, KlHxk1 (PDB ID: 3O08), (E) Dimeric archaeal ROK hexokinase PDB ID: 2E2N), (F) Tetrameric archaeal ROK hexokinase (PDB ID: 3VOV), (G) Tetrameric PvHK (current study). Individual protomers are shown in cyan, pink, purple and gold.
